# The association between body height and cancer: a retrospective analysis of 784,192 outpatients in Germany

**DOI:** 10.1007/s00432-022-04335-0

**Published:** 2022-09-06

**Authors:** Sarah Krieg, Christoph Roderburg, Andreas Krieg, Tom Luedde, Sven H. Loosen, Karel Kostev

**Affiliations:** 1 Clinic for Gastroenterology, Hepatology and Infectious Diseases, Medical Faculty of Heinrich Heine University Düsseldorf, University Hospital Düsseldorf, Moorenstrasse 5, 40225 Düsseldorf, Germany; 2 Department of Surgery (A), Medical Faculty of Heinrich Heine University Düsseldorf, University Hospital Düsseldorf, Moorenstrasse 5, 40225 Düsseldorf, Germany; 3 IQVIA, Frankfurt, Germany

**Keywords:** Tumor, Malignancy, Body composition, Incidence

## Abstract

**Purpose:**

Cancer risk is determined by numerous factors. Recently, body height has been linked to different cancer sites in different populations.

**Methods:**

This retrospective cohort study included 784,192 adult outpatients with available body height values from 2010 to 2020 using the Disease Analyzer database (IQVIA). The outcome was the incidence of cancer diagnoses within the study period according to body height, stratified by age, sex, and cancer sites.

**Results:**

Overall cancer incidence rose with increasing body height in both sexes. In women, there was a rise from 10.9 (≤ 160 cm) to 13.6 (> 180 cm) and from 16.6 (≤ 160 cm) to 26.8 (> 180 cm) cases per 1000 patient years in the 51–60 and > 70 years age group, respectively. Among men, cancer incidene increased from 23.9 (≤ 165 cm) to 26.3 (176–185 cm) and from 38.9 (≤ 165 cm) to 43.4 (176–185 cm) cases per 1000 patient years in 61–70 and > 70 years age group, respectively. The hazard ratio (HR) for developing cancer was 1.11 (95% CI 1.09–1.13) for every 10 cm increase in body height among women and 1.06 (95% CI 1.04–1.08) among men. A significant association between body height and cancer incidence was found for certain cancer sites, such as malignant melanoma, in both women (HR 1.21, 95% CI 1.11–1.33) and men (HR 1.29, 95% CI 1.18–1.42).

**Conclusion:**

In this study, we present the first data from a large cohort from Germany that provide strong evidence for a positive association between body height and the overall risk of developing various cancers.

## Introduction

Cancer is a leading cause of death worldwide, accounting for nearly 10 million deaths in 2020 (Ferlay et al. 2020). The most common causes of cancer deaths in 2020 were lung cancer (1.80 million deaths), colon and rectal cancer (916,000 deaths), liver cancer (830,000 deaths), stomach cancer (769,000 deaths), and breast cancer (685,000 deaths) (World Health Organization 2022). Cancer arises from the transformation of normal cells into tumor cells in a multistep process that generally develops from a precancerous lesion to a malignant tumor. These changes are the result of the interaction between a person's genetic factors as well as external influences such as physical carcinogens (e.g., ultraviolet and ionizing radiation), chemical carcinogens (e.g., asbestos, components of tobacco smoke, alcohol, aflatoxin, and arsenic), and biological carcinogens (e.g., infections caused by certain viruses, bacteria, or parasites). Recently, an association between obesity (or visceral obesity) and cancer has been suggested by many different authors (World Cancer Research Fund/American Institute for Cancer Research 2018; Avgerinos et al. 2019; Weihrauch-Blüher et al. 2019). In contrast, body height is less commonly reported to be linked to cancer risk (World Cancer Research Fund/American Institute for Cancer Research 2018; Choi et al. 2019). Based on the results of global cancer prevention and survival analyses, the 2018 World Cancer Research Fund (WCRF)/American Institute for Cancer Research (AICR) report stated that, in general, there is evidence that the taller people are during adulthood and the more people weighed at birth, the higher their risk for some cancers (World Cancer Research Fund/American Institute for Cancer Research 2018). Specifically, an increase in risk was found for a total of 8 cancer sites for each additional 5 cm of body height. Those cancers were cancer of the colorectum, breast, ovary, pancreas, endometrium, prostate, kidney, and malignant melanoma. In contrast, evidence for other cancer sites was discussed but was too limited to draw a conclusion: mouth, pharynx and larynx, esophagus (adenocarcinomas and squamous cell carcinomas), lung, stomach, gallbladder, cervix, and bladder (World Cancer Research Fund/American Institute for Cancer Research 2018). While different reports suggested increasing overall cancer rates with increasing body height, results for specific cancers at various anatomic sites including the breast, prostate, and colorectum (de Waard 1975; Albanes et al. 1988; Hebert et al. 1997) were inconsistent according to gender and/or body mass index (Batty et al. 2006, 2010; Sung et al. 2009; Green et al. 2011). Body height might be considered a marker or indicator of a number of events and experiences from childhood to adulthood (World Cancer Research Fund/American Institute for Cancer Research 2018). The increased risk that taller people have for different cancer sites and in different populations suggests that an underlying common mechanism might be involved. Given the lack of similar epidemiological analyses in Germany, we examined the IQVIA Disease Analyzer (DA) database for the association between body height and cancer risk using data from more than 780,000 patients from general practices (GPs).

## Methods

### Database

This study used data from the Disease Analyzer (DA) database (IQVIA), which has already been extensively described in the literature (Rathmann et al. 2018). To summarize, the DA database contains demographic, diagnostic and prescription data from patients followed in GP in Germany. Practices to include in the database are selected based on multiple factors (i.e., physician’s age, specialty group, community size category, and German federal state), and the database is composed of around 3–5% of all practices in Germany. Diagnosis and prescription data are coded using the International Classification of Diseases, 10th revision (ICD-10), and the Anatomical Classification of Pharmaceutical Products of the European Pharmaceutical Marketing Research Association (EphMRA), respectively. Finally, data are anonymously sent to IQVIA on a regular basis, and the quality of these data is assessed using several criteria such as completeness of documentation and linkage between diagnoses and prescriptions.

### Study population

In this retrospective cohort study, body height values were available for 830,916 (16.8%) of 4,945,928 individuals > 18 years (age at index date) followed in the 758 practices in Germany between January 2010 and December 2020. The only inclusion criteria was at least one documented body height value. The first body height value documented between January 2010 and December 2020 were considered index date. Individuals with a cancer diagnosis in the whole history prior to or at index date were excluded. A total of 784,192 individuals were finally available for analyses.

### Study outcomes and variables

The outcome of the study was the incidence of cancer diagnoses within the study period as a function of body height. Individuals were followed until cancer diagnosis, the last follow-up visit, or the end of the study period (May 31, 2022). Documented body height was measured by physicians and not self-documented. Body height was included as a four-category variable; for women: ≤ 160 cm, 161–170 cm, 171–180 cm, > 180 cm, and for men: ≤ 165 cm, 166–175 cm, 176–185 cm, > 185 cm. Cancer (ICD-10: C00–C97) diagnoses were analyzed separately for lip, oral cavity and pharynx (ICD-10: C00–C14), digestive organs (ICD-10: C15–C25 incl. esophagus (C15), stomach (C16), colon (C18), rectum (C20), anus and anal canal (C21), liver (C22), pancreas (C25)), respiratory organs (ICD-10: C30–C39 incl. larynx (C32) and bronchus and lung (C34)), skin (ICD-10: C33, C34 incl. melanoma (C33) and other skin cancers (C34)), female breast (ICD-10: C50), female genital organs (ICD-10: C51–C58), prostate (ICD-10: C61), urinary tract (ICD-10: C64–C67 incl. kidney (C64) and bladder (C67)), as well as lymphoid, hematopoietic and related tissue (C81–C95, including lymphomas (C81–C89), and leukemias (C91–C95)).

### Statistical analyses

Age at first visit was compared between height categories. As there was a strong relationship between body height and age (taller people were younger), all analyses were performed either by age group or adjusted for age. First, incidence of cancer by age group was shown for men and women. Then, the association between body height and cancer by sex were analyzed with Cox regression models adjusted for age at index date. The results of the Cox regression analyses are displayed as hazard ratios (HRs) and 95% confidence intervals (95% CI) for each cancer subtype. The HRs express how the risk of cancer increases by each 10 cm increase in height. Due to multiple comparison and high patient samples, only *p* values lower than 0.001 were considered statistically significant. All analyses were conducted with SAS 9.4 (SAS Institute, Cary, USA).

## Results

### Study cohort characteristics

Of 784,192 study patients, 415,396 patients were female with a mean age (SD) of 51.3 (18.3) years and mean body height (SD) of 164.8 (6.9) cm. Male patients (*n* = 368,796) had a mean age (SD) of 50.3 (17.4) years and a mean body height (SD) of 178.0 (7.5) cm. For women, most patients (*n* = 218,159) were categorized in the body height group of 161–170 cm. The majority of men (*n* = 172,054) were categorized in the body height group of 176–185 cm. Table 1 provides a detailed overview on the study cohort characteristics.

**Table 1:** Age at index date and body height of study patients with documented body height values

Variable	Women				
	Total	≤ 160 cm	161–170 cm	171–180 cm	> 180 cm
*N*	415,396	119,846	218,159	72,914	4477
Age at index date (mean, SD)	51.3 (18.3)	58.0 (18.7)	50.2 (17.7)	43.9 (15.6)	39.9 (13.5)
Height (mean, SD)	164.8 (6.9)	156.7 (3.4)	165.8 (2.7)	174.3 (2.7)	183.9 (2.8)

Variable	Men				
	Total	≤ 165 cm	166–175 cm	176–185 cm	> 185 cm
*N*	368,796	18,198	120,456	172,054	58,088
Age at index date (mean, SD)	50.3 (17.4)	60.5 (18.6)	54.6 (17.8)	48.5 (16.5)	43.1 (14.5)
Height (mean, SD)	178.0 (7.5)	162.3 (3.2)	171.6 (2.6)	180.1 (2.8)	189.5 (3.4)

### Incidence of cancer among different body height categories

**Fig. 1:**
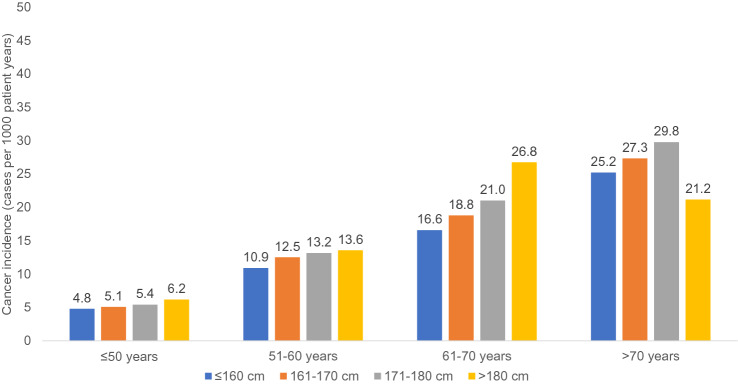
Incidence of cancer by age and body height categories among women

Among women in the age group ≤ 50 years, the incidence of cancer increased from 4.8 (≤ 160 cm) to 6.2 cases per 1000 patient years (> 180 cm), in the age group 51–60 years, from 10.9 (≤ 160 cm) to 13.6 cases per 1000 patient years (> 180 cm), and in the age group > 70 years, the incidence of cancer increased from 16.6 (≤ 160 cm) to 26.8 cases per 1000 patient years (> 180 cm, Fig. 1).

**Fig. 2:**
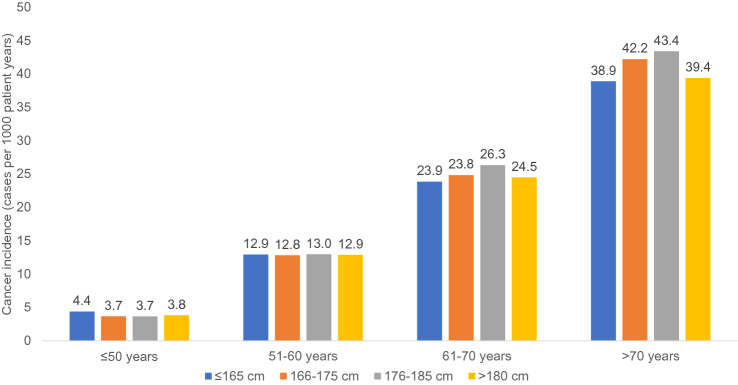
Incidence of cancer by age and body height categories among men

Among men, the incidence of cancer increased from 23.9 (≤ 165 cm) to 26.3 cases per 1000 patent cases (176–185 cm) in the age group 61–70 years, and from 38.9 (≤ 165 cm) to 43.4 (176–185 cm) in the age group > 70 years (Fig. 2). Of note, in men we did not observe a further increase in cancer incidence if the body height was over 185 cm (Fig. 2).

### Association between body height and different cancer sites

For cancer in total, the hazard ratio (HR) was 1.11 (95% CI 1.09–1.13, *p* < 0.001) for every 10 cm increase in body height among women and 1.06 (95% CI 1.04–1.08, *p* < 0.001) among men, respectively (Table 2). Looking at different cancer sites, a significant association between body height and cancer development among women was found for malignant melanoma (HR 1.21, 95% CI 1.11–1.33), breast cancer (HR 1.21, 95% CI 1.17–1.26), lymphomas (HR 1.11, 95% CI 1.05–1.18), and colon cancer (HR 1.17, 95% CI 1.08–1.27). In men, there was a significant association between body height and cancer risk for malignant melanoma (HR 1.29, 95% CI 1.18–1.42), prostata cancer (HR 1.15, 95% CI 1.10–1.19), and lymphoid, hematopoietic and related tissue cancer (HR 1.10, 95% CI 1.05–1.16) (Table 2). In contrast, there was no significant association between body height and cancer incidence with respect to cancers of the lip, oral cavity and pharynx, urinary tract or respiratory organ cancer for both women and men, as well as genital organ cancers among women, respectively (Table 2).

**Table 2:** Association between body height and cancer by sex and age (Cox regression models)

	Women		Men	
	Risk increase by 10 cm body size (hazrd ratio, 95% CI)	*p* value	Risk increase by 10 cm body size (hazrd ratio, 95% CI)	*p* value
Cancer total	1.11 (1.09–1.13)	< 0.001	1.06 (1.04–1.08)	< 0.001
Lip, oral cavity and pharynx	0.98 (0.82–1.16)	0.772	0.89 (0.79–1.00)	0.053
Digestive organs	1.08 (1.02–1.13)	0.004	0.99 (0.95–1.03)	0.498
Esophagus	0.95 (0.76–1.20)	0.663	0.97 (0.84–1.12)	0.676
Stomach	0.92 (0.80–1.06)	0.269	0.89 (0.79–1.01)	0.064
Colon	1.17 (1.08–1.27)	< 0.001	1.02 (0.95–1.10)	0.590
Rectum	1.02 (0.89–1.18)	0.735	1.07 (0.96–1.19)	0.229
Anus and anal canal	1.00 (0.67–1.47)	0.986	1.09 (0.76–1.57)	0.641
Liver	1.04 (0.84–1.28)	0.726	0.91 (0.79–1.04)	0.167
Pancreas	1.14 (1.01–1.29)	0.037	0.95 (0.85–1.07)	0.433
Respiratory organs	1.09 (1.01–1.17)	0.021	0.96 (0.91–1.02)	0.171
Larynx	1.26 (0.88–1.79)	0.205	0.96 (0.79–1.16)	0.641
Bronchus and lung	1.09 (1.01–1.18)	0.029	0.96 (0.91–1.02)	0.226
Skin	1.10 (1.06–1.15)	< 0.001	1.11 (1.07–1.15)	< 0.001
Melanoma	1.21 (1.11–1.33)	< 0.001	1.29 (1.18–1.42)	< 0.001
Other skin cancer	1.08 (1.03–1.13)	0.001	1.07 (1.03–1.12)	0.001
Breast	1.21 (1.17–1.26)	< 0.001	–	
Female genital organs	1.08 (1.01–1.15)	0.109	–	
Prostate	–		1.15 (1.10–1.19)	< 0.001
Urinary tract	0.99 (0.90–1.09)	0.838	1.04 (0.99–1.11)	0.143
Kidney	1.03 (0.89–1.18)	0.725	1.05 (0.95–1.16)	0.345
Bladder	0.89 (0.76–1.04)	0.134	1.04 (0.95–1.13)	0.372
Lymphoid, hematopoietic and related tissue	1.05 (1.00–1.11)	0.065	1.10 (1.05–1.16)	< 0.001
Lymphomas	1.11 (1.05–1.18)	< 0.001	1.10 (1.04–1.17)	0.001
Leukemias	0.93 (0.82–1.05)	0.217	1.13 (1.02–1.25)	0.017

## Discussion

In this study, for the first time in a large cohort in Germany, we examined cancer incidence as a function of adult body height, stratified by sex, age, and cancer type, using data from the IQVIA DA database of a total of 784,192 outpatients in 758 GP. Consistent with previous studies, we found evidence that taller body height is positively associated with an increased risk of developing certain malignancies. Of note, this association was found in both sexes. Interestingly, the largest body height effect was found among individuals aged 70 and above, in both men and women. Our data indicate that the overall risk of cancer increases significantly per 10 cm body height in both sexes. Remarkably, the relationship between body height and cancer incidence seems to be stronger among women. Looking more closely at specific cancer sites, body height was most strongly associated with malignant melanoma incidence in both women and men. In contrast, no significant association was found between body height and cancer incidence for lip cancer, pharyngeal cancer, urinary tract cancer or respiratory organ cancer in either sex. There was also no association between body height and the incidence of female genital organs. The association of cancer risk with body height has already been studied in different populations depending on different cancer sites (World Cancer Research Fund/American Institute for Cancer Research 2018). By analyzing nationwide claim data of 22,809,722 Korean participants including both men and women (2009–2012), Choi et al. demonstrated that within 765,651 patients who developed cancer during a 5-year follow-up period, body height was positively associated with risk of all site-combined cancers and with malignancies in the oral cavity, larynx, lung, stomach, colorectum, liver, pancreas, biliary tract, gallbladder, breast, ovary, cervix, corpus uteri, prostate, testes, kidney, bladder, central nervous system, thyroid, skin, and lymphatic or haematopoietic systems (Choi et al. 2019). Similarly, in the “Million Women Study”, an analysis including 1,297,124 women who were followed up for a total of 11.7 million person-years, cancer incidence rised with increasing adult body height for most cancer sites (Green et al. 2011). As a possible explanation for the association between body height and cancer incidence, it has been hypothesized that as body height increases, there are also more cells, including stem cells, exposed to a higher risk of oncogenic somatic mutations. In this context, a multistage model of carcinogenesis has been described, according to which cancer arises from the accumulation in a cell of a cancer-specific series of driver mutations, which may be a mixture of DNA mutations, epigenetic reprogramming, and/or chromosomal alterations (Craig et al. 1987). Accordingly, the probability of cancer developing depends on the number of dividing cells, the number of divisions each cell line undergoes, the somatic mutation rate, as well as the number of driver mutations required. An alternative hypothesis discussed in the literature is that the association between body height and cancer incidence is indirect only, given that the biological determinants of body height are multifactorial. According to this, body height is influenced by various factors in early life such as nutrition, psychosocial stress and infectious diseases (Gunnell et al. 2001; Batty et al. 2006; Watters et al. 2009). Furthermore, factors such as the level of growth-determining hormones, particularly Insulin-Like Growth Factor 1 (IGF-1), are thought to play an important role in the size effect (Gunnell et al. 2001; Kabat et al. 2014). Thus, the level of circulating IGF-1 has been shown to correlate positively with skeletal growth in children as well as with height in adults (Hawley et al. 1989; Key et al. 2010; Crowe et al. 2011). Interestingly, lower cancer rates have been observed in individuals with Laron syndrome, a rare autosomal recessive inherited disease with growth hormone receptor mutation (GHRD) in which affected individuals have decreased serum IGF-1 levels and severely reduced body stature with an average adult height of approximately 118 cm in females and 124 cm in males (Laron et al. 1993). In particular, for malignant melanoma, the strong size effect on cancer incidence is hypothesized to be caused by the higher cell division rate and increased IGF-1 levels. As a function, IGF-1 has been shown to stimulate proliferation of keratocytes, fibroblasts, and other skin cells, which could influence skin cancer risk (Conover et al. 1983; Barreca et al. 1992; Cats et al. 1996; Nunney 2018). The fact that certain cancers exhibit no significant association with body height may be partly explained by the effect of body height being masked by other significant dominant risk factors. According to this, Albanes et al., also failed to demonstrate a size effect in oral and pharyngeal cancers, which are strongly associated with smoking (Albanes 1998). This observation is also consistent with the results of previous studies demonstrating that the association with body height was lower for smoking-related cancers than for others (Green et al. 2011; Kabat et al. 2013). Thus, in the Women's Health Initiative Study, lung cancer incidence was more strongly related to height in never-smokers than in current or former smokers. For cancers of the female genital organs, the size effect might be masked by the fact that cervical cancer is strongly associated with HPV infection (Bosch et al. 2002). However, because our study design yields only associations and not causalities, the question of why some cancers exceptionally do not have an apparent association with body height remains conclusively unclear and needs to be further investigated in future prospective trials.

Height in adulthood is associated with genetic birth weight, growth rate, and age at puberty, as well as environmental factors. Similarly, infant growth may be able to be accelerated by feeding a high-protein diet, which in turn is expected to result in increased adult body height. Increases in body height can be expected if the population is less susceptible to malnutrition and infection. The influence of the environment might be evident in the increase of about 1 cm per decade in body height in the European population during the twentieth century, which led Green et al. to conclude that this could explain an increase of about 10–15% in cancer prevalence over the years (Cavelaars et al. 2000; Garcia et al. 2007; Green et al. 2011).

However, some limitations should be acknowledged when interpreting this study´s results. First, our study is subject to the inevitable limitations of a longitudinal and retrospective analysis of a large database. It should be noted that our data are exclusively descriptive. Secondary data analyses such as the present study are typically limited by the incompleteness of the underlying data. All diagnoses were documented with ICD-10 codes, potentially leading to misclassification and undercoding of certain diagnoses. For example, coding of female genital organs does not distinguish between cancer of the ovary, cervix, and endometrium, despite clear evidence from the WCRF that body size is strongly associated with endometrial and ovarian cancer but not with cervical cancer (World Cancer Research Fund/American Institute for Cancer Research 2018). Furthermore, no documentation was provided on how and where the cancer diagnoses were made as GPs often use letters from hospitals or specialists for the documentation of diagnoses.

Specifically, our study focused on body height, tumor diagnosis, age and sex. In contrast, there were no information on socioeconomic status, environmental conditions or life-style factors (e.g., nicotine and alcohol consumption), that would have allowed more detailed analyses, so matching patients with these potential confounders was not feasible. Although the results of our study are supported by the findings of previous prospective cohort studies, no causal relationships but only associations can be concluded. Nonetheless, the strength of our study was the large number of patients included and the use of data representative of GP in Germany. Since the range of body height in a given population is usually narrow, a large number of events is required for reliable risk estimation. Conclusively, the IQVIA DA database used for the analyses in this study has been extensively validated in several studies and has been shown to be representative of the outpatient sector (Becher et al. 2009; van den Boom et al. 2020; Roderburg et al. 2021).

## Conclusion

Taken together, our results from a large German cohort provide strong evidence that the risk for certain but not all cancer sites rises with increasing body height. These associations were observed for both sexes and in every age group. Knowledge of how adult body height affects risk for specific cancer sites could support the development of interventions to improve health throughout the life course worldwide. However, further research, such as on hormones and other growth factors related to body height, is needed to definitively address the question of whether taller individuals should be provided risk screening for certain cancer sites.

## Data Availability

The datasets used and/or analyzed during the current study are available from the corresponding author on reasonable request.
